# A new methodology to structure a nutrition educational course: the results of a short-term educational-intervention pilot study

**DOI:** 10.1017/jns.2023.55

**Published:** 2023-07-06

**Authors:** Maria Vittoria Conti, Marco Gnesi, Noemi Zelaschi, Kassandra Yaghi, Layal Ghazi, Hellas Cena

**Affiliations:** 1 Laboratory of Dietetics and Clinical Nutrition, Department of Public Health, Experimental and Forensic Medicine, University of Pavia, Via Bassi 21, 27100 Pavia, Italy; 2 Section of Biostatistics and Clinical Epidemiology, Department of Public Health, Experimental and Forensic Medicine, University of Pavia, 27100 Pavia, Italy; 3 Department of Nutrition and Dietetics, Faculty of Health Sciences, Beirut Arab University, P.O. Box 11-5020 Riad El Solh, Beirut 11072809, Lebanon; 4 Clinical Nutrition and Dietetics Service, Unit of Internal Medicine and Endocrinology, ICS Maugeri IRCCS, 27100 Pavia, Italy

**Keywords:** Africa, Education, Food knowledge, Triple burden of malnutrition, Women

## Abstract

The incidence of malnutrition in developing countries, such as Kenya, is still high. The most recent international reports outline an increase in the prevalence of overnutrition in Sub-Saharan Africa, where undernutrition and hidden hunger conditions still represent an emergency. Among the factors influencing malnutrition conditions connected to dietary intake and eating habits, evidence shows the importance of food knowledge. Traditionally, in developing countries, women represent the pillar of the food system and impact their children's nutrition: food-based, women-centred strategies are recommended to address nutrient gaps, educate and empower women, and counteract malnutrition. The short communication presents the results of the ACTION project: an educational-intervention pilot study targeting childbearing-age women living in rural areas of Southern Kenya. To evaluate the feasibility of the educational course built, the authors administered to the women a food knowledge questionnaire, before and after the intervention. Study results describe an increase in women's food knowledge after attending the food education course. This encourages to increase education courses which can represent a starting point in improving food habits with a positive effect on the health condition in developing counties too.

## Introduction

Food Knowledge (FK) is defined as the ‘individual understanding of basic information about food and nutrition’, influencing dietary intake and eating habits with consequences to human health^(1)^. According to recent literature results, the nutritional status of young children in developing countries is improved by nutrition education given to their caregivers^(2)^: higher levels of caregivers’ FK are associated with increased consumption of fruits and vegetables and reduced consumption of nutrient-poor, energy-dense foods by children^(3)^.

In Kenya, an increase in FK among women has been positively associated with nutritional outcomes in their children^(4)^. Moreover, personalised, home-based maternal nutrition counselling, administered during pregnancy and one year after delivery, reduced children's stunting prevalence in the first and fifth years of life, resulting in better feeding and hygiene practices and less morbidity^(5)^. Accordingly, a nutritional educational intervention targeting women could be fundamental to counteract the malnutrition condition. The latter still represents a looming problem in the global epidemiological scenario.

In 2020, the World Health Organization redefined malnutrition and classified it into three different categories of conditions: undernutrition, micronutrient-related malnutrition and overnutrition^(6)^. Recently, in developing countries, a significant shift in the dietary pattern, known as Nutrition Transition, led to a coexisting triple burden of malnutrition^(7)^. This transition is a consequence of the globalisation of the food system^(7)^. Diets started shifting towards an increased reliance on processed, ready-made, cheaper meals, increased away-from-home food intake and increased use of edible oils and sugar-sweetened beverages^(8)^, which has led to a growing rise in non-communicable diseases (NCDs).

For instance, Kenya has witnessed a decreased prevalence of undernutrition: the stunting rate in children under 5 years of age decreased from 33 % in 2000 to 22 % in 2020. From 2000 to 2019, the underweight rate also decreased in the adult population^(9)^. In contrast, the prevalence of overweight and obesity in Kenyan adolescents and adults is constantly increasing^(9)^. Those trends of double burden conditions also characterised the female population in developing countries^(10)^, leading to a greater risk of dietary deficiency^(11)^.

Poor maternal nutrition is also associated with maternal disease, mothers’ lack of infection resistance and poor maternal and perinatal outcomes^(12)^. Therefore, especially among women of childbearing age (WCA), guaranteeing good health is essential for them and also for the health of their unborn children^(11)^. This is mainly evident in developing countries, such as Sub-Saharan Africa, where inadequate micronutrient intake causes detrimental effects on all people, particularly children and WCA^(13)^.

In these countries, women are traditionally the primary caregivers of the family and main providers of food, having both a direct and indirect influence on their children's nutrition through their childcare habits and their own nutritional status, caring for the family and prioritising the wellbeing of the children^(13)^.

For this reason, a woman-centred education and empowerment paradigm is advised in order to achieve long-term and more sustainable benefits for the entire community^(13)^.

Several actions have been taken to improve the nutritional status among the Kenyan general population, such as taxes on sugar-sweetened beverages and strategies to reduce unhealthy diets and NCDs^(9)^. Other strategies include iron, folate, vitamin A and zinc supplementation^(14)^. Despite that, as described above, recent trends show high prevalence rates of malnutrition among the Kenyan population.

Therefore, in a socio-political and agricultural strategy tackling malnutrition aimed at WCA at different levels, a further building block could be an educational process that provides greater awareness and autonomy in food choice by enhancing FK.

The objective of this educational-intervention pilot study, named *nAkuru, kenya: local seed valorisation and nutritional eduCaTION (ACTION)*, is to evaluate the feasibility of a target-specific population educational model for improving the food and agricultural knowledge of the population living in the Nakuru District (Kenya).

## Materials and methods

The educational-intervention pilot study, ACTION project, was carried out in three villages in the Nakuru District (Kenya): *Karai, Makongo and Wanyororo*.

To evaluate how the FK level of participants changed before and after the intervention, the authors administered a modified version of the previously validated Food Knowledge Questionnaire (FKQ)^(15)^. The FKQ consists of eighty-eight items divided into ten different sections (from A to L), each exploring various aspects of the FK. Starting from this structure, a series of project-specific questions were added as Section M. The latter is derived from a specific validated questionnaire aimed at evaluating the FK of women in relation to food habits and consumption during pregnancy and feeding time^(16)^.

The recruitment occurred in March 2022 (baseline, T0), before the educational activity.

Women were interviewed again after the educational intervention, in May 2022 (follow-up, T1).

The live interviews were conducted in Swahili by a trained local enumerator of the Slow Food Kenya team. The enumerator administered the questionnaires through face-to-face interviews, physically going to the three different villages where the women, recruited into the study, lived. All villages were located in the Nakuru District, Kenya, and a recruitment point was established in each village. Established in 2014, Slow Food Kenya is an affiliate of Slow Food International, a global, grassroots organisation founded in 1989 to prevent the disappearance of local food cultures and traditions, counteract the rise of ‘fast life’, and combat people's dwindling interest in the food they eat.

The nutrition educational activities were administered through video pills recorded in K-Swahili. The topics were selected based on a literature research related to further food education interventions aimed at women in Sub-Saharan Africa and the lack of knowledge that characterised this target population. The main reference for the video contents was the National Guidelines for Healthy Diets and Physical Activity, released by the Kenyan Ministry of Health in 2017^(17)^.

The study sample included WCA (15−49 years old) living in the Nakuru district (Kenya). Participants were consecutively selected (convenience sampling), and each respondent of legal age signed a written informed consent; for minors, parents or legal guardians were asked to sign the consent forms. This study was conducted according to the Declaration of Helsinki.

According to the exploratory nature of this pilot study, which was mostly intended as a feasibility, no formal *a priori* sample size estimation was done.

The statistical analysis was based on descriptive methods. The sample was described in terms of socio-demographic variables (Section A of the questionnaire). Quantitative variables were described as medians and interquartile ranges (IQRs), while qualitative variables were described as counts and percentages. The number of missing data was assessed, but no data imputation was made.

FKQ scores were computed according to the criterion proposed in the validation study of the Tanzanian FKQ^(14)^. For each FK Section (B to L), the Section's score was computed by summing 1 for each correct answer, 0 otherwise. A total FKQ score was then derived from the Sections’ scores by counting the number of sections where the respondent scored above the sample median for that Section. The same scoring procedure was applied for baseline scores (T0) and post-intervention scores (T1), but reference medians were always those observed at T0.

Scores at T0 and T1 for Sections B to L were described in terms of medians and IQRs at T0 and T1. The share of participants in which the score decreased, remained unchanged, or increased from T0 to T1 was described as counts and percentages. Items in Section M were described individually as counts and percentages.

When applicable, the significance threshold for inferential testing was set at 0⋅05; however, significance must be interpreted cautiously, according to the pilot nature of the study.

Analyses were conducted using Stata 13.1 (StataCorp. 2013. Stata Statistical Software: Release 13. College Station, TX: StataCorp LP).

## Results and discussion

The primary outcomes of this pilot study are the implementation of the educational intervention and the feasibility assessment of the educational model created within the context of the ACTION project. Seven video pillars, covering a variety of topics regarding nutrition in the life course, each of 15 min, made up the educational activities.

As supplemental material, two educational brochures were handed out at the end of the study to provide women with more information on the subjects covered during the training.

This pilot study enrolled fifty WCA living in the villages of Karai, Makongo and Wanyororo (Nakuru District). A description of the socio-demographic characteristics of the sample (Section A) is reported in Table 1. The median age of respondents was 35 years old (IQR 12 years), where all women were Christian. The majority of the women in the sample completed primary or secondary education (72⋅0 %). Almost all women reported having a job (96⋅0 %), most frequently ‘farmer’ (66⋅7 %), ‘food business’ (6⋅3 %) and ‘agribusiness’ (4⋅2 %). More than half of the women were married (64⋅0 %) and three-quarters of the sample had at least one child (88⋅0 %).

**Table 1.** Socio-demographic characteristics of the sample

Current region, *n* (%)	Nakuru	47 (94⋅0)
	Karai	1 (2⋅0)
	Rift valley	2 (4⋅0)
Marital status, *n* (%)	Single	(26⋅0)
	In a relationship	(6⋅0)
	Married	(64⋅0)
	Widow	(2⋅0)
	Divorced	(2⋅0)
Number of children, median (IQR)		3 (4)
Religion, *n* (%)	Christian	50 (100⋅0)
Schooling level, *n* (%)	None	1 (2⋅0)
	Primary	25 (50⋅0)
	Secondary	13 (26⋅0)
	High School	2 (4⋅0)
	Technical/Tertiary	1 (2⋅0)
	Diploma	4 (8⋅0)
	Degree	3 (6⋅0)
	Post Graduate	1 (2⋅0)
Have a job, *n* (%)	None	2 (4⋅0)
	Yes	48 (96⋅0)
Kind of job, *n* (%)	Agribusiness	2 (4⋅2)
	Farmer	32 (66⋅7)
	Self-employed	2 (4⋅2)
	Food business	3 (6⋅3)
	Policewomen	1 (2⋅1)
	Teacher	1 (2⋅1)
	Student	1 (2⋅1)
	Tailor	1 (2⋅1)
	Vegetable and fruit vendor	1 (2⋅1)
	Poultry farmer	1 (2⋅1)
	Nutritional consultant	1 (2⋅1)
	Business	1 (2⋅1)
	Hairdresser	1 (2⋅1)
Control on food expenditure, *n* (%)	Mother	16 (32⋅0)
	Father	31 (62⋅0)
	Other	1 (2⋅0)
	Mother and Father	2 (4⋅0)
Following a diet, *n* (%)	Yes	2 (4⋅0)
	Not	48 (96⋅0)

To verify the impact of the intervention on the FK sample, the number (and percentage) of women who answered correctly or incorrectly in each Section (from B to M), at T0 and T1, were compared and described in Table 2.

**Table 2.** Items scores at baseline (T0) and after the educational intervention (T1)

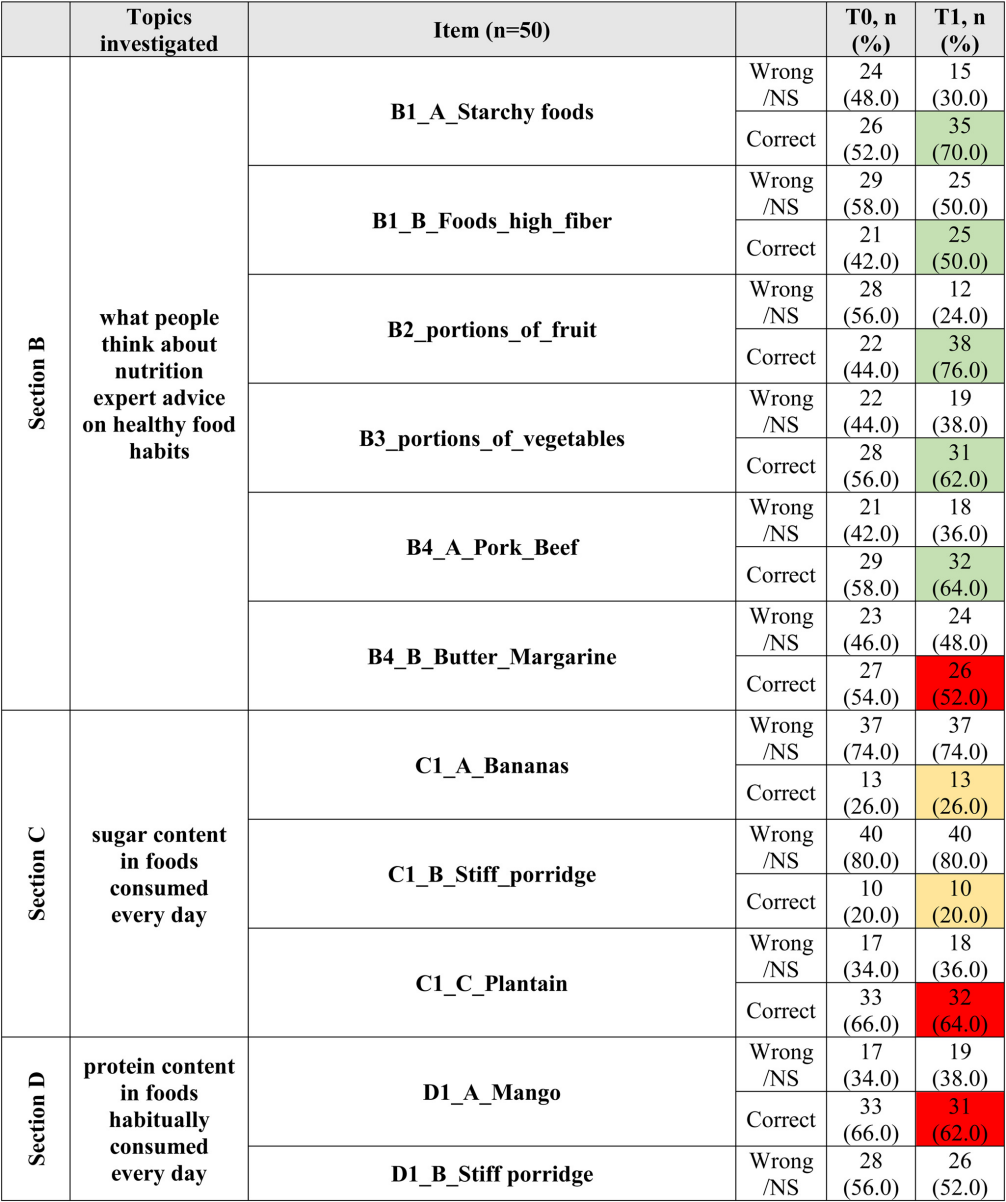
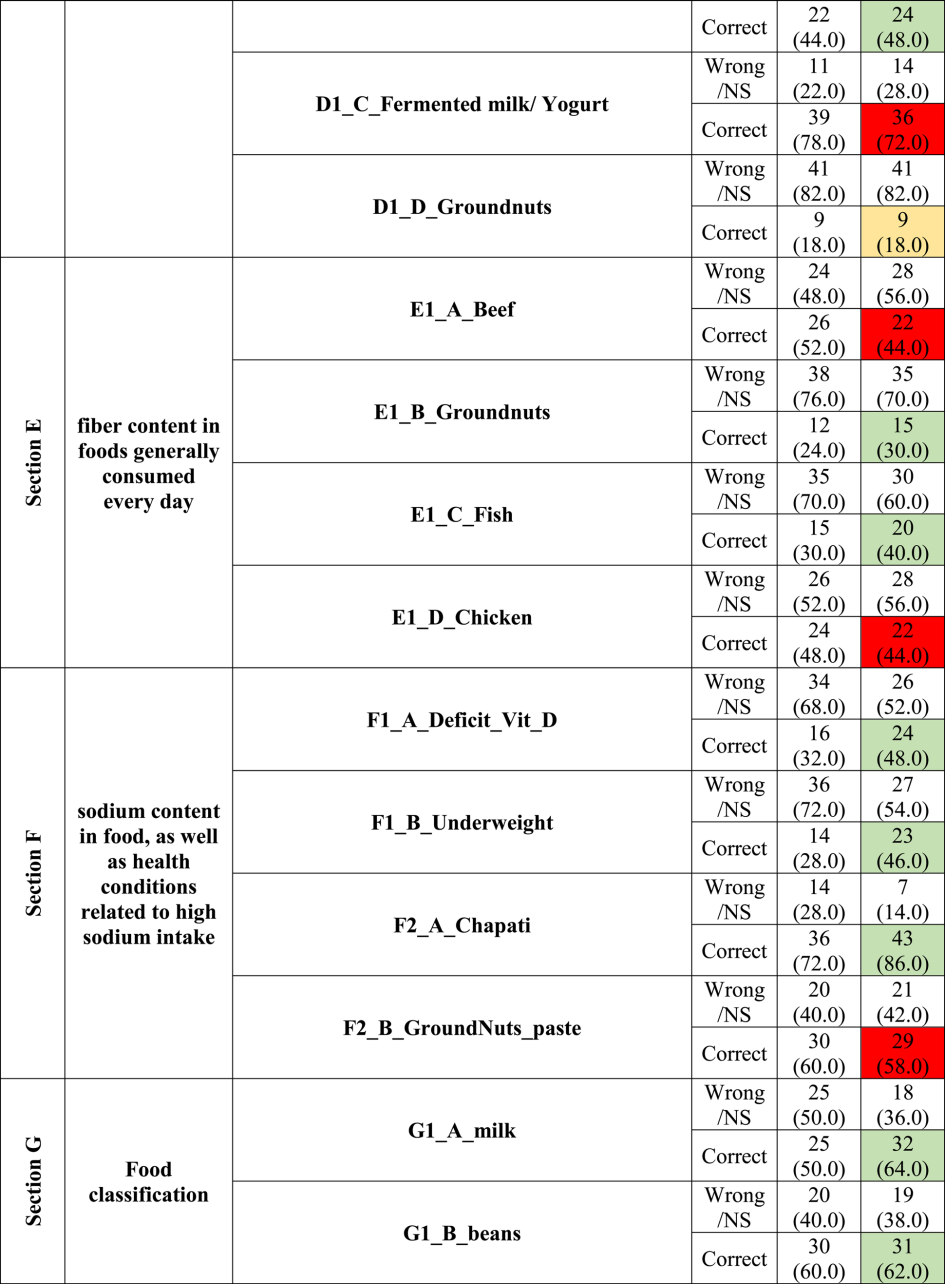
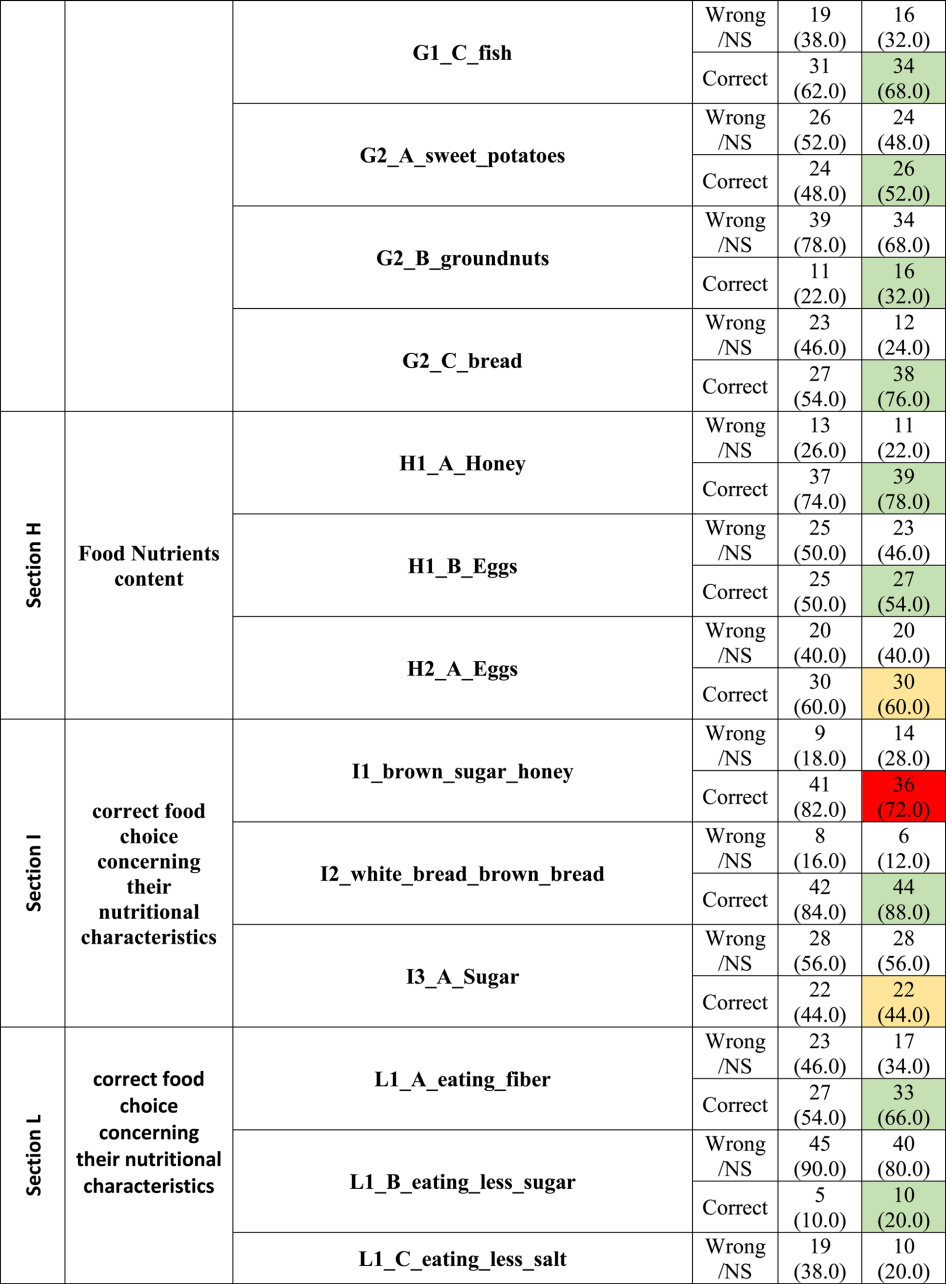
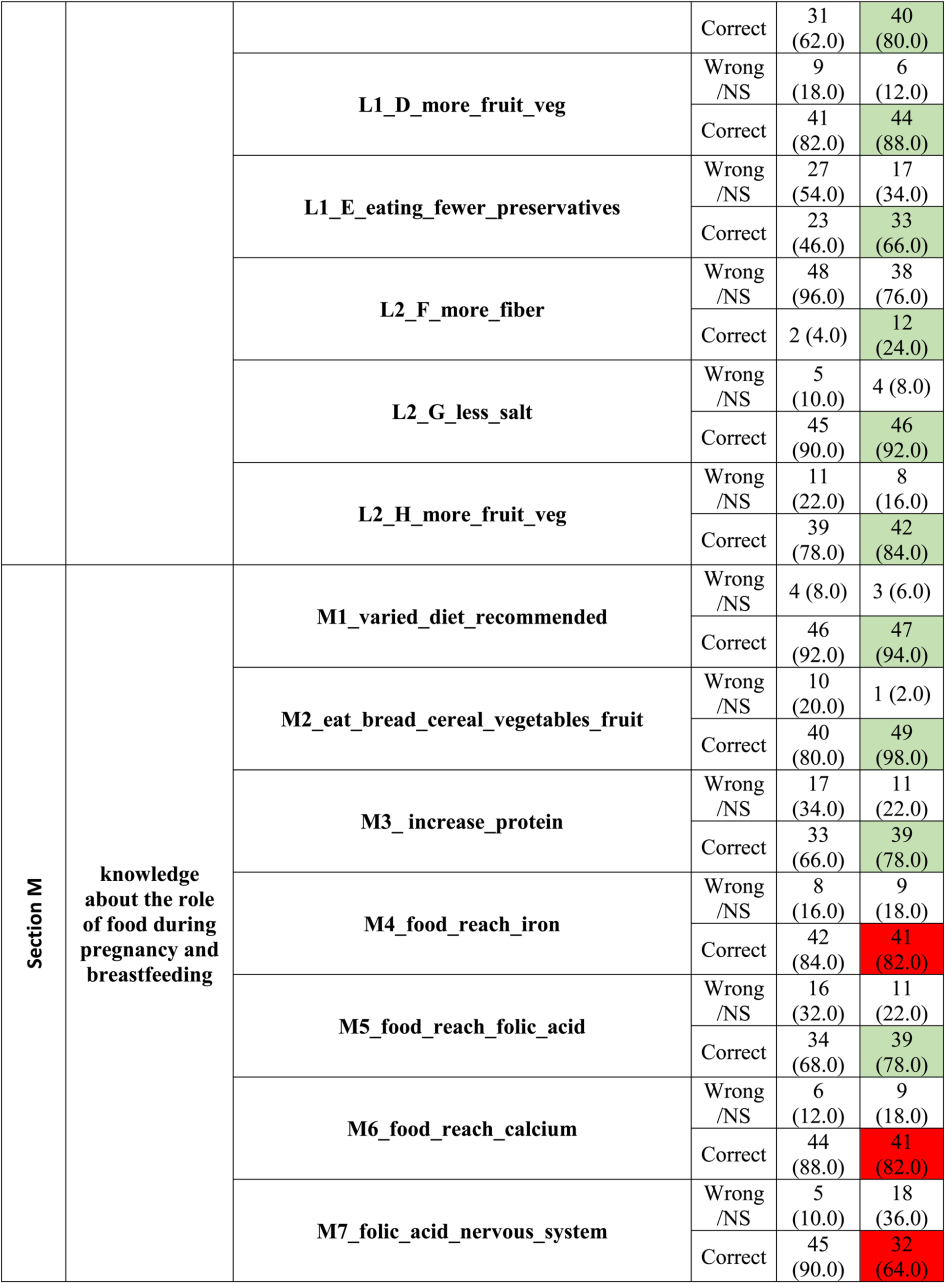
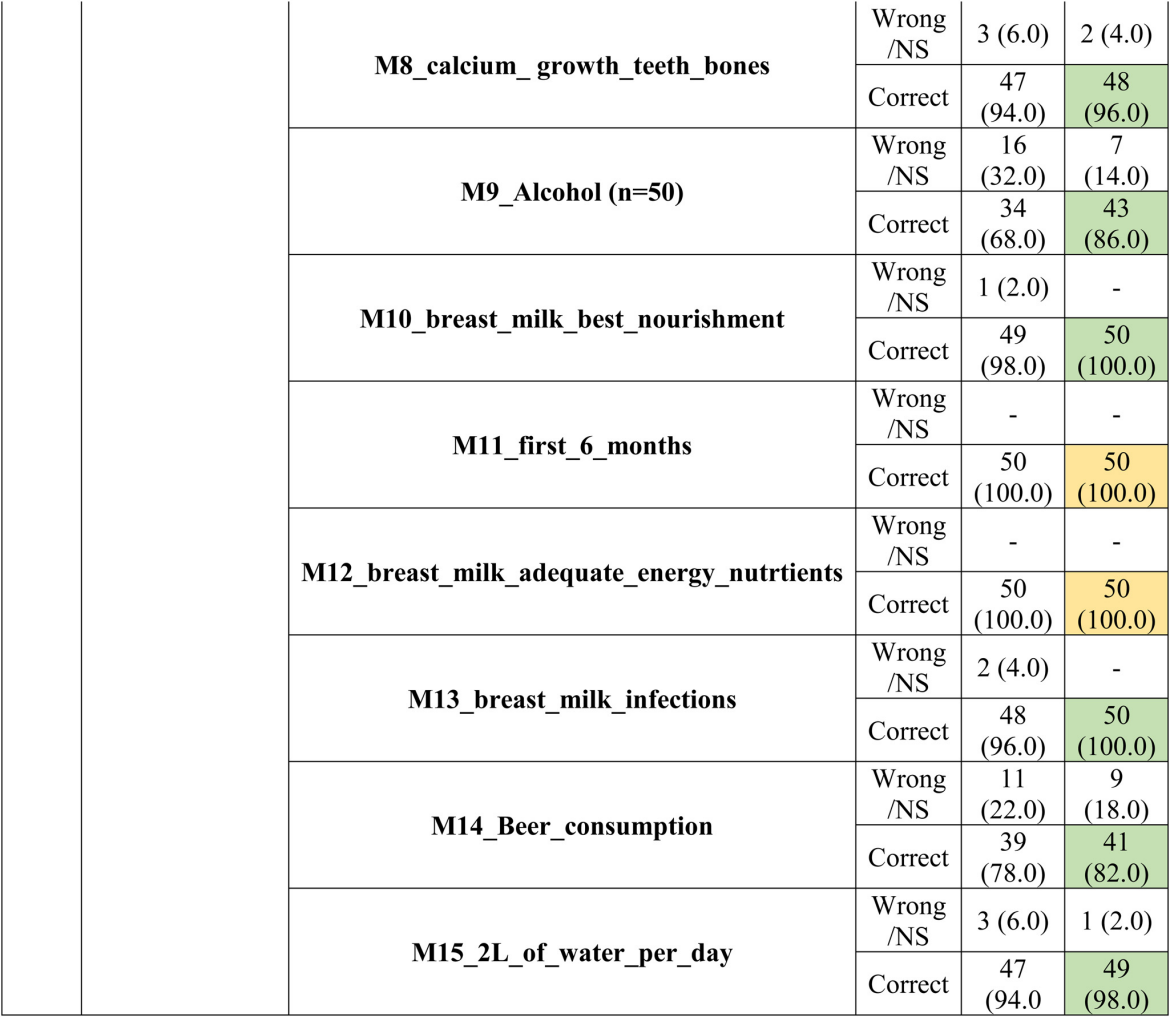

Colour legend: green represents an improvement from T0 to T1 (i.e. the number of correct answers increased); yellow indicates no change was observed from T0 to T1; red represents a worsening from T0 to T1 (i.e. the number of correct answers decreased).

Section B showed an improvement in almost all the questions except one regarding the reduction in margarine/butter consumption. In Sections C and D, no improvement was observed between T0 and T1: in question C1-C (sugar content in the Plantain), a worsening was noted. The same happened in questions D1-A (protein content in mango) and D1-C (protein content in yogurt).

Questions E1-A and E1-D recorded a worsening in the answer about fibre content in beef and chicken. In Section I, a worsening was observed in the item which investigated whether brown sugar was healthier than honey.

On the other hand, for Sections F, G, H and L, a general and consistent trend of improvement between T0 and T1 was registered.

Looking at the scores of each FKQ Section, reported in Table 3, there was a trend of improvement in FK in Sections B, E, F, G and L; the difference between scores was statistically significant for Sections B and L. When looking at the Total FKQ Score, the median value increased by 0⋅5 points from T0 to T1, and the difference was not statistically significant; a score increase was observed in 16 respondents (32⋅0 %), while in the remaining 34 (68⋅0 %), the score either decreased or remained unchanged.

**Table 3.** Scores of FKQ sections and total FKQ score at baseline (T0) and after educational activities (T1)

	T0	T1	Test T0 *v.* T1		Increase
	*Median (IQR)*	*Median (IQR)*	*z*	*P*-value	*n* (%)
Section B	3 (2)	4 (2)	−2⋅082	0⋅037	14 (28⋅0)
Section C	1 (1)	1 (1)	−0⋅172	0⋅864	12 (24⋅0)
Section D	2 (1)	2 (2)	0⋅115	0⋅908	16 (32⋅0)
Section E	1⋅5 (3)	2 (3)	−0⋅054	0⋅957	17 (34⋅0)
Section F	2 (2)	3 (1)	−1⋅851	0⋅064	15 (30⋅0)
Section G	3 (4)	4 (3)	−1⋅775	0⋅076	14 (28⋅0)
Section H	2 (2)	2 (2)	−0⋅229	0⋅819	19 (38⋅0)
Section I	2 (1)	2 (1)	0⋅261	0⋅794	13 (26⋅0)
Section L	5 (3)	6 (1)	−3⋅087	0⋅002	10 (20⋅0)
Total FKQ score	6⋅5 (3)	7 (3)	−1⋅650	0⋅099	16 (32⋅0)

The number of respondents whose scores increased from T0 to T1 is reported in the last column.

IQR, interquartile range.

Concerning Section M, an improvement for most of the questions is described, except for questions M-4, M-6 and M-7.

This manuscript focuses on the results of the nutritional activities of the ACTION project, designed to target WCA, acknowledging their central role in the Food System^(13)^ as supported by many recent studies conducted in Kenya, which found that women's FK has been positively associated with nutritional outcomes in children^(4)^.

The feasibility of the present study indicates a positive impact based on the results of the FKQ where shares of correct answers were found to increase for many items (Table 3). This indicates a trend of improvement in several domains of FK after the administration of the education program (T1) compared to the baseline (T0).

However, some items found a worsening or non-change in the share of correct answers from T0 to T1. The reason behind the lack of improvement can be due to unclear information (e.g. Section E1-A/D) not treated in the video pillars (e.g. Section B4-B), or a misunderstanding from the women (e.g. Section M4/6/7).

The interventional course was delivered to the population online in seven brief video pills, lasting 15 min each. This education model finds various points of difference with respect to the results described in previous literature: the way to administer the course, its duration and the topics addressed.

Concerning the way of administration, the majority of other studies used ‘conventional techniques’ such as frontal and face-to-face lessons in studies conducted in Western Kenya^(18)^ and rural China^(19)^, training manuals and posters in a study in Turkana (Kenya)^(20)^, charts, brochures and models in a study in Kwale district of Kenya^(21)^.

Regarding the duration, the nutritional educational course of the ACTION project was condensed: all the seven video pillars were administered within 1 week. Literature results highlight the effectiveness of a time-focused intervention in low-income groups due to unemployment, transport issues, rural residence and an unstable state of mental health^(22)^.

The results of other studies described a longer average duration of the educational activities, as is the case in the aforementioned study in Western Kenya, where the education intervention lasted 4 months^(18)^.

As for the topics covered in the educational videos of the ACTION project, different aspects of FK were tackled: food composition, the role of micronutrients and macronutrients, the relationship between nutrition and health in the lifecycle and the prevention of NCDs. These topics were partially similar to those used in other studies: for example, in the Kwale district, Hitachi *et al.* focused on maternal nutrition during pregnancy and lactation, or on the importance of exclusive breastfeeding and child nutrition^(21)^.

To verify the feasibility of the study, the authors started from a validated FKQ and adapted it to the Kenyan culture and dietary habits. It was then used to describe the women's FK score variation before and after the intervention^(1)^.

Also, an adapted version of a validated questionnaire developed in Indonesia to evaluate maternal FK was used by Wx *et al.* in Turkana, Kenya^(20)^. Not all studies used a validated tool, which is the case in Western Kenya, where nutritional knowledge was assessed using a score range of 0–21 through open questions^(18)^ and in the Kwale district of Kenya, where caregivers’ attitude on FK was assessed based on nine items corresponding to the tailored educational guidelines^(21)^.

In almost all mentioned studies, an improvement in the population knowledge was noticed: in particular, in the study of Waswa *et al.*^(18)^, where children's dietary scores and caregivers’ nutrition knowledge scores improved significantly. In the study of Mbogori *et al.*, maternal knowledge on the duration of exclusive breastfeeding, the recommended composition of complementary foods and understanding of the importance of feeding food to a child with diarrhoea improved significantly after the intervention^(20)^. Results from Zhang *et al.* also indicate that an educational intervention can enhance caregivers’ knowledge and practices of complementary feeding and ultimately improve children's growth^(19)^.

The key advantages of the ACTION study are the use of a structured, adapted, previously validated population-specific questionnaire^(15)^, as well as the method used to create the intervention, its administration through distance education, its duration and its focused and condensed design. Moreover, the intervention also featured lectures and brochures as additional resources among its many other components.

Also, all the research assistants were adequately trained by the investigators before data collection in order to ensure data integrity.

Despite these strengths, there are some limitations. First of all, the small sample size due to the pilot nature of the study and the absence of a control group.

Therefore, results should be interpreted cautiously, as a ‘proof of concept’, and further research will be needed in order to properly assess the efficacy of the remote intervention.

The lack of a ‘practical section’ in the context of the education course can represent a further limitation in understanding the contents and establishing the new concepts acquired through the training.
